# Complete genome sequence of invasive *Streptococcus pneumoniae* serotype 11A causing meningitis in an adult patient, Italy 2022

**DOI:** 10.1128/mra.00054-24

**Published:** 2024-04-08

**Authors:** Stefano Amadesi, Caterina Vocale, Davide Guariglia, Monica Cricca, Tiziana Lazzarotto, Vittorio Sambri, Paolo Gaibani

**Affiliations:** 1 IRCCS Azienda Ospedaliero-Universitaria di Bologna, Bologna, Italy; 2 Department of Medical and Surgical Sciences, Alma Mater Studiorum, University of Bologna, Bologna, Italy; 3 Department of Diagnostics and Public Health, Microbiology Section, Verona University, Verona, Italy; University of Maryland School of Medicine, Baltimore, Maryland, USA

**Keywords:** gram positive, invasive infection, cerebrospinal fluid, *Streptococcus pneumoniae*, Italy

## Abstract

*Streptococcus pneumoniae* is a major global health concern, being a common cause of meningitis in both children and adults. Here, we report the complete genome sequence of P10_PNE_LCR, a *S. pneumoniae* 11A strain isolated in Northern Italy from an adult patient diagnosed with meningitis.

## ANNOUNCEMENT

*Streptococcus pneumoniae* is a major cause of meningitis in children and adults (1, 2). Infections caused by this pathogen often lead to neurological complications in survivors, including hearing impairment and cognitive deficits (3). Serotype identification of circulating strains is important to reduce cases of invasive bacterial disease that can be prevented by vaccination. In Italy in 2022, serotype 11A resulted among the 12 most prevalent serotypes circulating in adults. Here, we describe the complete genome of P10_PNE_LCR, a *S. pneumoniae* belonging to serotype 11A responsible for a case of meningitis in an adult patient. The P10_PNE_LCR strain was isolated on 13 February 2023 from the cerebrospinal fluid of a 64-year-old female patient hospitalized at Maurizio Bufalini Hospital in Cesena, Italy. Species identification was performed by MALDI-TOF (Bruker Daltonics, Germany) mass spectrometry. The strain was cultured on Blood Agar plate overnight at 37°C. Genomic DNA was isolated from cell cultures using the Dneasy Blood & Tissue Kit (Qiagen, Switzerland). Illumina and Nanopore libraries were prepared using the Illumina DNA Prep Kit and the Nanopore Rapid Sequencing Kit V14 (SQK-RAD114) with no prior size-selection and according to the instructions provided by the respective manufacturers. Whole-genome sequencing was performed on both Illumina iSeq 100 (Illumina, USA) and Nanopore MinION Mk1C (Flow Cell R9.4.1) (Oxford Nanopore Technologies, UK) systems. Nanopore base calling was carried out locally using MinKNOW v22.12.5 software. Read quality assessment was performed for both Illumina and Nanopore sequences using FastQC v0.12.1 (4). Genome assembly was executed based on Nanopore reads with Flye v2.9.2 (parameters --iterations 2 --genome-size 2 m) (5), and the obtained sequence was polished using Illumina reads with Polypolish v0.5.0 (6). Genome circularity was confirmed with Bandage v0.8.1 (7). No sequence trimming or rotation was performed. Multi-locus sequence type was determined with MLST v2.23.0 (https://github.com/tseemann/mlst). Capsular type was assigned using PneumoCaT v1.2.1 (8). Global Pneumococcal Sequence Cluster (GPSC) type was assessed using PopPUNK v2.6.0 (9). The genome was annotated with RASTtk v4.0 (10) and with PGAP pipeline according to the information on number of predicted genes. Antimicrobial resistance and virulence genes were detected with Abricate v1.0.1 (https://github.com/tseemann/abricate) and further inspected with BLAST v2.15.0 (11). Default parameters were used except where otherwise noted. Illumina sequencing produced a total of 1,280,226 paired-end reads with a length of 151 bp, while Nanopore sequencing yielded 15,499 reads ranging from 102 to 55,291 bp in length, average length 3,652 bp, and N50 9,155. The genome was composed of a single circular contig of 2,027,961 base pairs, with 39.78% G + C content and 123× coverage. Genome-based typing demonstrated that P10_PNE_LCR belonged to sequence type ST62, serotype 11A, lineage GPSC 5. Sequence analysis of antimicrobial resistance genes revealed that the strain carried genes associated with resistance against fluoroquinolones (*patA*, *patB*, *pmrA*) and macrolides (*rlmA*). Moreover, several determinants of virulence were detected, including genes involved in adhesion (*cbpC*, *cbpD*, *cbpG*, *pavA*, *pavB*, *pcpA*, *pspA*), cell wall hydrolysis (*hysA*, *lytA*, *lytB*, *lytC*, *pytD*), protection against phagocytosis (*cps4A*, *cps4B*), pilus formation (*pitA*, *pitB*, *sipA*, *srtG1*, *srtG2*), production of toxins (*ply*), ion-binding proteins (*piaA*, *piuA*, *psaA*), zinc metalloproteases (*cppA*, *htrA*, *iga*, *tig*, *zmpB*, *zmpC*), neuraminidases (*nanA*, *nanB*, *nanC*), and enolases (*eno*).

## Data Availability

The genome assembly of strain P10_PNE_LCR has been deposited in the NCBI Genbank Database under accession number CP136899.1. Sequencing reads have been deposited in the NCBI SRA Database under accession numbers SRR27587389 and SRR27587390.
